# Explicit Ion Effects on the Charge and Conformation of Weak Polyelectrolytes

**DOI:** 10.3390/polym11010183

**Published:** 2019-01-21

**Authors:** Vikramjit S. Rathee, Hythem Sidky, Benjamin J. Sikora, Jonathan K. Whitmer

**Affiliations:** Department of Chemical and Biomolecular Engineering, University of Notre Dame, Notre Dame, IN 46556, USA; vrathee@nd.edu (V.S.R.); hsidky@uchicago.edu (H.S.); bsikora@kcp.com (B.J.S.)

**Keywords:** weak polyelectrolytes, complexation, polyelectrolyte complexation, coil-globule transition, polyelectrolyte brushes, phase transition, separations, drug delivery

## Abstract

The titration behavior of weak polyelectrolytes is of high importance, due to their uses in new technologies including nanofiltration and drug delivery applications. A comprehensive picture of polyelectrolyte titration under relevant conditions is currently lacking, due to the complexity of systems involved in the process. One must contend with the inherent structural and solvation properties of the polymer, the presence of counterions, and local chemical equilibria enforced by background salt concentration and solution acidity. Moreover, for these cases, the systems of interest have locally high concentrations of monomers, induced by polymer connectivity or confinement, and thus deviate from ideal titration behavior. This work furthers knowledge in this limit utilizing hybrid Monte Carlo–Molecular Dynamics simulations to investigate the influence of salt concentration, ${\mathrm{pK}}_{a}$, pH, and counterion valence in determining the coil-to-globule transition of poorly solvated weak polyelectrolytes. We characterize this transition at a range of experimentally relevant salt concentrations and explicitly examine the role multivalent salts play in determining polyelectrolyte ionization behavior and conformations. These simulations serve as an essential starting point in understanding the complexation between weak polyelectrolytes and ion rejection of self-assembled copolymer membranes.

## 1. Introduction

Polyelectrolytes are the basis for a broad class of functional systems including self-assembled copolymer membranes, polymer-coated nanoparticles, polyelectrolyte microgels, and hydrogel networks [1,2,3,4,5,6,7,8,9,10,11]. Though regarded as innately charged, polyelectrolytes frequently contain *weakly* acidic or basic moieties distributed along the polymer backbone. This can be immensely useful, as monomer groups may protonate or deprotonate depending upon the local environmental conditions, enabling tunable “smart” materials [12,13,14]. Important recent examples include the pH-responsive swelling and collapse of poly(acrylic acid) [PAA] in functionalized nanoporous membranes [15], pH-responsive Saloplastics/Compact polyelectrolyte complexes (COPECs) [16] synthesized using Poly(methacrylic acid) (PMAA) poly(allylamine hydrochloride) (PAH), which find applications in tissue engineering by mimicking mechanical properties of a cartilage [17], self-assembled polyelectrolyte capsules and multilayer thin films for drug delivery purposes [18,19,20].

Polyelectrolyte materials in the form of coacervates have been of great interest recently, both for their uses within the food processing industry [21,22]. As well as their role in underwater adhesives and encapsulation [21,23,24]. Extensive studies on the effects of counterions in determining the assembly and stability of polyelectrolyte complexes have been performed by several groups [25,26,27,28,29,30,31,32,33], though these have largely ignored the role of solution acidity in determining the charge on these compounds, which is crucial for dense polyelectrolyte assemblies [28,34,35,36,37,38]. Importantly, it was recently shown that the pH could significantly alter the charging equilibrium, and indeed the underlying thermodynamics of the process, when acidity and salinity are explicitly accounted for [33]. Many of these effects derive from the physical proximity of weakly charging groups to each other. The effects of polyelectrolyte density can be particularly prominent when there are many charged species present, such as when weak polyelectrolytes are realized as brushes adhering to a larger molecule or surface, which have been examined for applications in pH-responsive sensors [39], drug delivery complexes [40], and nanoparticle matrices [41]. The ionization of weak monomers in brush layers differs significantly from that in bulk, with acid groups away from a substrate’s surface proving easier to ionize than those closer to the surface, with an overall decreasing charge fraction as the thickness of the brush is increased [42,43]. Strong evidence exists from simulations to explain these effects through the instantaneous environment of each titratable monomer, which is affected by the presence of other ions, polyacids and polybases in the vicinity [33,44,45,46,47,48].

In particular, for single-chain systems, it has been demonstrated both experimentally [49,50,51,52] and computationally [46,53,54,55,56] that the well-known transition of a poorly solvated titratable polymer from a collapsed (globule) state to an extended (coil) state (the coil–globule transition, or CGT) as a function of pH can be strongly dependent on the salt content of a solution. The CGT in particular, is of great interest in drug delivery, as the transition from a loaded globule state to an expanded coil can release drug at a specific site of action defined by the local pH [24]. Interactions between the polyelectrolyte molecule and dissolved ions are key to this behavior on two fronts. First, higher salt concentrations lead to increased screening, which reduces repulsion from the charging of bound monomers. The extent of this effect depends on the structure of the polymer, as topologically enforced monomer densities limit the effects of screening, and shifts the polyelectrolyte charge away from that expected naively from monomer titration experiments [35,45,57,58]. Second, strongly coupled ions (whether through multivalence or low-dielectric solvent conditions) can bind to the polyelectrolyte, thus affecting its conformations and local charging characteristics. Still, despite the prevalence of weakly acidic or basic polymers in various applications within the polyelectrolyte literature, there remains relatively little understanding of how different effects (including pH, salt concentration, topology, and solvation) balance in determining polyelectrolyte structure, morphology, and function.

Emerging applications of weak polyelectrolytes in smart materials demand sensitive response, with systems balanced on a knife’s edge ready to adapt their microstructure precisely in response to the local stimuli, it is imperative that we be able to accurately understand the structure of weak polyelectrolytes across a wide range of acidity and salinity. While some theoretical work has been performed in the area of weak polyelectrolyte brushes [36,37,59,60,61], computer simulations, which often utilize simplified, coarse-grained models to extract essential trends and behavior, represent an ideal place to deepen understanding and develop intuitions for weak polyelectrolyte materials. For instance, simulations have been performed to elucidate the ionization of weak polyelectrolytes in non-aqueous environments [58], where it was found that ionization of weak polyelectrolytes may be enhanced by an increase of the electrostatic coupling constant $\Gamma =\lambda_{B}/\sigma$ (where $\lambda_{B}$ is the Bjerrum length and σ the size of monomers—see Section 2 for further discussion) due to energetically favorable interactions between a dissociated ion and monomer pair. Further complexation studies examining weak polyacids and nanoparticles in simulation have been performed in a wide array of circumstances, to uncover effects related to polyelectrolyte chain length, polyacid-to-nanoparticle mixing ratio [62] and ionic screening [44] on the ionization of the weak polyelectrolyte systems. In the case of weak polyacid–nanoparticle complexation, a study performed by Stornes et al. [62] found that for large polyacid-to-nanoparticle ratios and long chain lengths the titration curves exhibited multiple inflection points due to competition between polyacid-nanoparticle attractive interactions and the repulsive interactions among the like-charged monomers on the same and different polyacid chains. In a similar study, pairs of weak polyacids and polybases were seen to facilitate charging to take advantage of favorable electrostatic interactions [33,44]. There, it was found that complexation between weak polyacid and weak polybase may be induced through associative charging at low screening conditions corresponding to low monovalent salt concentration. Additional work has also examined the conformation of nanoparticle coronas formed due to electrostatic interaction between weak polyelectrolyte chains as functions of pH and solvent quality, where weak polyelectrolyte grafted nanoparticles were found to exhibit significant differences in morphology when compared to fixed charge models [57].

Here, we build on an initial study which examined the titration of weak polyelectrolyte chains and stars using coupled reaction Monte Carlo and Molecular Dynamics in an implicit-salt screened-charge interaction limit [45], by explicitly accounting for the ionic species via grand-canonical Monte Carlo coupling to salt and base reservoirs. This initial study observed significant and nontrivial topology-dependent effects on the charging behavior of polyelectrolytes with variable numbers of arms, but equal molecular weight. Though results were in good qualitative agreement with experiments on weak star polymers, the use of implicit ions limited the applicability of this description to monovalent (1:1) salts at low-to-moderate concentrations. There, ions can be assumed to be well-dispersed, and the effects of polymer–ion correlations on acid dissociation are limited. Explicit ion effects should contribute prominently to the ion–polymer association and charging behavior, particularly for multivalent species or in non-aqueous solvents where strong forces can lead to counterion adsorption onto the weak polyelectrolyte.

## 2. Methods

**Figure 1:**
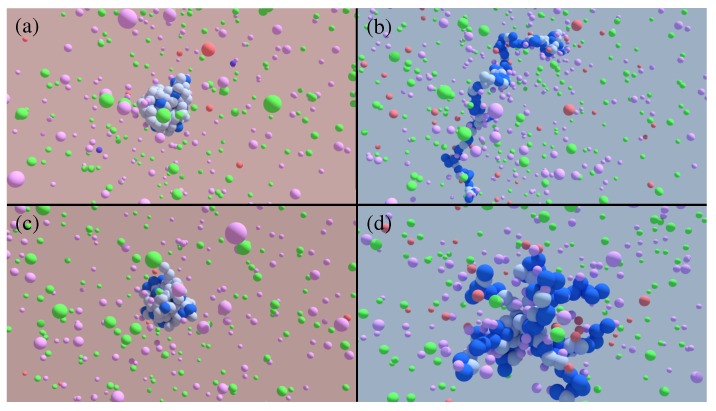
Typical simulation snapshots for linear and star polyelectrolytes on either side of the CGT. The red background denotes more acidic conditions ($\mathrm{pH}-{\mathrm{pK}}_{a}=1.302$) while the blue background denotes more basic conditions ($\mathrm{pH}-{\mathrm{pK}}_{a}=3.040$). Between these two states, a swelling transition exists whose sharpness is topology-dependent. States (**a**,**c**) correspond to collapsed globule configurations in the linear (**a**) and star (**c**) polyelectrolytes, with states (**b**,**d**) defining the corresponding swollen configurations. The salt concentration is regulated by chemical equilibrium with a solution of NaCl at 0.1 M concentration. Na${}^{+}$ and Cl${}^{-}$ are represented by pink and green beads, respectively. The titration steps (see methods for full description) require OH${}^{-}$ ions, which are regulated by chemical equilibrium with a reservoir of KOH. The pOH is fixed at ≈2.7 (pH≈11.3), with K${}^{+}$ ion being depicted in red and OH${}^{-}$ ion depicted in purple.

Here, we examine polyelectrolyte titration effects in the presence of explicit ions using a version of the restricted primitive model [63] and its extension to polymers [54] which is explicitly coupled to chemostats fixing the bulk salinity and pH. Figure 1 depicts typical coarse-grained linear and star-shaped weak polyelectrolytes along with ambient salt ions forming a charge cloud around the polyelectrolyte. These configurations are typical of the simulation setup utilized in this study, and the swelling observed when transitioning from more acidic (red) to more basic (blue) conditions.

Titration simulations in the presence of salt require a delicate balance of chemical and physical influences be maintained, and thus require a specific thermodynamic ensemble, augmented by specialized Monte Carlo moves. Here, we perform hybrid Molecular Dynamics and Monte Carlo simulations (MCMD) using LAMMPS [64] and SAPHRON [65]. The coupling incorporates multi-species grand canonical sampling along with reaction-ensemble moves to accurately capture the charging of a weak polyelectrolyte under dilute conditions. All simulations contain a single bead–spring polymer chain, of length N=121, having a coarse-grained bead size σ, in a linear or a 10-arm star configuration, which has arms of length $N_{\mathrm{arm}}=12$ emanating from a single central bead (see Figure 1). A periodic simulation box of fixed size ${\left(64.6\sigma \right)}^{3}$ was utilized for these simulations.

The total potential energy within a configuration of our system is a sum of charge interactions, bond potentials, and monomer adhesions and repulsions.

(1) $$U\left(x\right)=U_{\mathrm{LJ}}\left(x\right)+U_{\mathrm{FENE}}\left(x\right)+\;U_{\mathrm{coul}}\left(x\right)\;.$$

Here, *x* refers to all of the atomic coordinates in the system. Each of the terms in the equation above is a sum over pairwise interactions between interacting sites *i* and *j*. The first term is a shifted–truncated Lennard-Jones potential with a strength $\epsilon_{ij}=k_{B}T$ utilized for all interactions. The total Lennard-Jones interaction, accounting for these parameters, can be represented as:

(2) $$\begin{array}{ccc} U_{\mathrm{LJ}}^{ij}\left(r_{ij}\right) & = & 4\epsilon_{ij}\left[{\left(\frac{\sigma_{ij}}{r_{ij}}\right)}^{12}-{\left(\frac{\sigma_{ij}}{r_{ij}}\right)}^{6}\right] \end{array}$$

(3) $$\begin{array}{ccc} U_{{\mathrm{LJ}}_{\mathrm{trunc}}}^{ij}\left(r_{ij}\right) & = & \left\{\begin{array}{cc} U_{\mathrm{LJ}}^{ij}\left(r_{ij}\right)-U_{\mathrm{LJ}}^{ij}\left(r_{\mathrm{cut}}\right), & r_{ij}<r_{\mathrm{cut}}^{ij}\\ 0, & \mathrm{elsewhere}\; \end{array}\;\right.. \end{array}$$

Here $r_{ij}=x_{i}-x_{j}$ defines the separation vector between atoms *i* and *j*, and $r_{ij}$ defines its magnitude. While, in general the $\sigma_{ij}$ terms will change for different species, within this paper we consider the case where all $\sigma_{ij}$ are set to the same value, defining the reduced unit of length in our coarse-grained system. The cutoffs, however, are species dependent. Monomer–monomer interactions have an attractive well to stabilize the collapsed state. This serves to model polymers such as poly(2-vinylpyridine) (P2VP), poly(acrylic acid) (PAA) and poly(vinyl amine) (PVAm), which have been explored extensively in experiments [51,66,67] and have nominal adhesive interactions through hydrophobicity or hydrogen bonding when neutralized [68,69,70,71]. Thus, for the monomer–monomer interactions, $U_{{\mathrm{LJ}}_{\mathrm{trunc}}}^{ij}\left(r_{ij}\right)$, the cutoff distance was set to $r_{\mathrm{cut}}^{ij}=2.5\sigma$. Ion–ion and monomer–ion interactions are not presumed to have specific attractions, and thus utilize the same $\epsilon_{ij}$ as above, with a reduced cutoff distance $r_{\mathrm{cut}}^{ij}=2^{1/6}\sigma$, representing a purely repulsive interaction. When charged interactions are relevant, they are handled via the Ewald sum of the Coulomb potential [72],

(4) $$U_{\mathrm{coul}}^{ij}\left(r_{ij}\right)=k_{B}T\lambda_{B}\frac{q_{i}q_{j}}{r_{ij}}\;,$$

where $q_{i}\in \left\{-1,0,+1\right\}$ depending on the current chemical identity of atom *i* and $r_{ij}$ has units of σ, which is the reduced units of length in our simulations. These simulations utilize a cutoff distance of 18σ and set the dimensionless accuracy in the calculated electrostatic forces to be $10^{-5}$.

Finally, in keeping with prior work on bead–spring models, bonds are handled via a finitely-extensible nonlinear elastic (FENE) potential [54,73,74],

(5) $$U_{\mathrm{FENE}}^{ij}\left(r_{ij}\right)=0.5KR_{0}^{2}\log\left[1-{\left(\frac{r_{ij}}{R_{0}}\right)}^{2}\right]\;,$$

where $R_{0}=2\sigma$ and $K=7\epsilon_{ij}/\sigma$ represent maximum extent and spring constant, respectively [54]. The same parameters are used for star and linear polymers, with the central bead in the star connected via additional FENE springs to the base of each arm. This is sufficient to define all molecular interactions within the system. What then remains is to set the scales of interaction to best match the experimental systems of interest. Here, we assume that the titratable sites are linked to a standard polycarbonate backbone, and thus choose a Bjerrum length [75,76] as $\lambda_{B}=\frac{e^{2}}{4\pi \varepsilon k_{B}T}$=2.8σ, to match the typical length in aqueous media if σ is set to the repeat unit length of polyethylene, ≈0.25 nm. For this article, we perform simulations with $\lambda_{B}$ = 2.8σ, setting the charge coupling constant $\Gamma =\lambda_{B}/\sigma$ of 2.8. Finally, the masses $m_{i}$ of all species and the thermal energy scale were set to 1.0 in reduced units.

Now that we have described the model interactions, we proceed to describe our handling of chemical equilibria. Titration of the weak polyelectrolyte was performed using Reaction Ensemble Monte Carlo (RxMC) moves, a well-established method for simulating chemical equilibrium [77,78,79], to model (de)protonation of a weak polyacid.

(6) $$M^{-}⇌MH+OH^{-}$$

**Figure 2:**
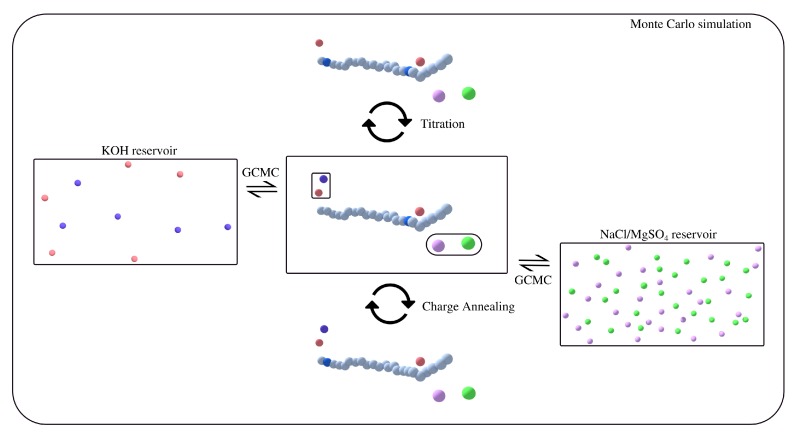
Schematic of the Monte Carlo moves utilized in our simulations. The color scheme is as follows: light blue and dark blue beads represent uncharged and charged monomers; red and purple beads represent K${}^{+}$ and OH${}^{-}$ ions; and pink and green beads represent Na${}^{+}$ and Cl${}^{-}$. The KOH and NaCl or MgSO${}_{4}$ reservoir are representative of bulk concentrations whereas the concentration within the simulation box is representative of the environment around the weak polyelectrolyte in the combined system. KOH concentration in the reservoir or bulk concentration is fixed at 0.002 M which corresponds to pOH ≈ 2.7 whereas the salt bulk concentration is considered here as a variable. The salt bulk concentration depicted in this schematic corresponds to ≈0.01 M. Titration moves charge the monomer bead, as defined in the governing reaction (Equation (6)). Charge annealing moves simulate the equilibrium dissociation and recombination of the monomer bead in another location on the polymer, while grand canonical Monte Carlo (GCMC) moves govern the exchange of ions with the bulk.

For simulations performed here, the deprotonation process (see Figure 2) involves removal of OH${}^{-}$ from the simulation box and an identity change for a randomly selected uncharged polyacid bead MH to that of the charged polyacid bead (M${}^{-}$). The probability of acceptance for a given deprotonation step is

(7) $$P_{\mathrm{acc}}=\min\left\{\Lambda^{3}{\left(\frac{m_{0}m_{M^{-}}}{m_{MH}\;m_{OH^{-}}}\right)}^{3/2}\frac{N_{MH}N_{OH^{-}}}{V\left(N_{M^{-}}+1\right)}e^{-\beta \Delta U+\beta \mu_{\mathrm{rxn}}},1\right\}\;,$$

where ΔU is the total change in potential energy from the force field, and $\mu_{\mathrm{rxn}}$ is the reaction chemical potential, the Gibbs energy associated with a single such reaction taking place. This is phrased so that a larger positive value of $\mu_{\mathrm{rxn}}$ favors deprotonated states where the polymer is charged. The protonation process involves addition of ${\mathrm{OH}}^{-}$ to the simulation box and changing the identity of randomly selected charged polyacid bead (M${}^{-}$) to that of uncharged polyacid bead MH. Hence, the probability of acceptance of this step is given by

(8) $$P_{\mathrm{acc}}=\min\left\{\Lambda^{-3}{\left(\frac{m_{MH}\;m_{OH^{-}}}{m_{0}m_{M^{-}}}\right)}^{3/2}\frac{VN_{M^{-}}}{\left(N_{MH}+1\right)\left(N_{OH^{-}}+1\right)}e^{-\beta \Delta U-\beta \mu_{\mathrm{rxn}}},1\right\}\;.$$

Selection of a deprotonation or protonation step is random. It is important to note that the charged and uncharged beads are treated as otherwise identical. Thus this change contributes to ΔU in two ways: (1) a change in the Coulomb energy due to the change in the bead’s charge state and (2) through the addition or removal of charge-balancing moieties. $\Lambda =\sqrt{h^{2}/2\pi m_{0}k_{B}T}$ is the de Broglie thermal wavelength for a particle of mass $m_{0}$ and mass of all species is set equal to 1.0 in reduced units. The term $\mu_{\mathrm{rxn}}$, which appears in each of these expressions, can be related to experiment by noting

(9) $$\mu_{\mathrm{rxn}}=\ln\left(10\right)\left[\mathrm{pH}-{\mathrm{pK}}_{a}\right]-\mu_{OH^{-}}\;,$$

where ${\mathrm{pK}}_{a}$ is the $-\log\left(K_{a}\right)$ of the polyacid monomer, and $\mu_{OH^{-}}$ is the chemical potential of the OH${}^{-}$ ions. This definition is similar to the one used in a previous investigation by Barr and Panagiotopoulos [57] for understanding the conformation transitions of weak polyacids grafted to nanoparticles. Varying the values of $\ln\left(10\right)\left[\mathrm{pH}-{\mathrm{pK}}_{a}\right]$ in these titration moves can be thought of as the sweeping of the dissociation constant $K_{a}$ for acid monomers, and thus is equivalent to a change in the chemical nature of the monomer beads in a given environment. Along with the titration moves, we allow charges on the polymer to anneal via species swap moves accepted according to the standard Metropolis criterion [45,72]. This facilitates swift relaxation of the conformational degrees of freedom.

Finally, to regulate salt concentrations, we utilize grand canonical Monte Carlo (GCMC) moves that have been implemented for the exchange of salt (NaCl or MgSO${}_{4}$) and buffer solution (KOH) between the simulation box and reservoir at a fixed chemical potential corresponding to a known bulk concentration obtained through independent salt-only GCMC simulations. The GCMC moves for salt species (NaCl or MgSO${}_{4}$) are independent from those for KOH, and the order of attempts is randomly selected to maintain detailed balance. Acceptance rates for these moves are given by

(10) $$P_{\mathrm{insertion}}=\min\left\{\prod_{i=1}^{k}\left[\frac{V}{\Lambda^{3}\left(N_{i}+1\right)}e^{\beta \mu_{i}}\right]e^{-\beta \Delta U},\;\;1\right\}$$

for the insertion move, and

(11) $$P_{\mathrm{deletion}}=\min\left\{\prod_{j=1}^{k}\left[\Lambda^{3}\frac{N_{j}}{V}e^{-\beta \mu_{j}}\right]e^{-\beta \Delta U},\;\;1\right\}\;.$$

noindent for deletion moves. Here, $\mu_{i}$ and $\mu_{j}$ are the chemical potentials of the species labeled *i* and *j* that the GCMC moves are performed on, whereas $N_{i}$ and $N_{j}$ correspond to the number of these particles prior to performing the move, with *k* representing the number of ion species participating in the current multi-species GCMC move. For consistency, results are presented in this article in terms of charging chemical potential, $\mu =\ln\left(10\right)\left[\mathrm{pH}-{\mathrm{pK}}_{a}\right]$. For the simulations where explicit salt effects are investigated, the chemical potential of the salt is varied to achieve different bulk salt concentrations. For the purposes of reporting data, we represent the effects of salt through the Debye screening length $\lambda_{D}$,

(12) $$\lambda_{D}=\frac{1}{\sqrt{8\pi \lambda_{B}I}}\;.$$

Here, I corresponds to the ionic strength (in moles per liter) of the salt reservoir, obtained by monitoring the average occupancy of a pure-salt box at the same chemical potential as the polymer simulations. We use this mapping to compare results using explicit salt to those obtained using the implicit Debye-Hückel model [45]. All parameters in the two models are identical, though the reaction moves are significantly simplified in the implicit simulations since an explicit representation of the base is not required [48].

The chemical potential of KOH is fixed for all simulations and corresponds to a bulk concentration ≈0.002 M (pOH≈2.7; pH≈11.3). A chemostat regulates the supply of ${\mathrm{OH}}^{-}$ for RxMC moves. The K${}^{+}$ and Na${}^{+}$ ions have identical interactions in our system but are handled as independent entities in our implementation. It is necessary to utilize this high OH${}^{-}$ concentration to render explicit-ion simulations computationally tractable while still retaining enough OH${}^{-}$ in the box to participate in reaction moves with reasonable frequency. For example, at pH=pOH=7, the simulation box would need to be ≈${\left(1150\sigma \right)}^{3}$ to have a similar number of OH${}^{-}$ ions in our simulations, rendering experimentally relevant salt concentrations on the order of 0.01–0.1 M prohibitively expensive. However, we would like to note that with sufficiently advanced computational resources, the methodology presented here could be directly applied at arbitrary pH through scaling the simulation box.

The configurations obtained from Monte Carlo simulations are relaxed via short MD simulations, with timestep size δt=0.01τ in reduced units and $\tau =\sigma \sqrt{m_{0}/\epsilon_{\mathrm{LJ}}}$. A Langevin thermostat was utilized to maintain the temperature at a value of 1.0 in reduced units. The MD simulations consist of 1000 steps (1 MD sweep), and 30 MC moves per MD sweep were attempted, distributed equally among RxMC, GCMC and Charge Annealing moves. The total number of Monte Carlo moves performed in each simulation was $\approx 7\times 10^{5}$, whereas the total number of molecular dynamics steps was $\approx 2\times 10^{7}$.

## 3. Results

With the model in place, we proceed to examine several variables that affect the overall charge state of the polyelectrolyte and the resulting CGT behavior. When discussing our results, we refer to the monovalent and divalent salts as NaCl and MgSO${}_{4}$ respectively, though the only chemical difference we take into account is the valence of cation and anion.

**Figure 3:**
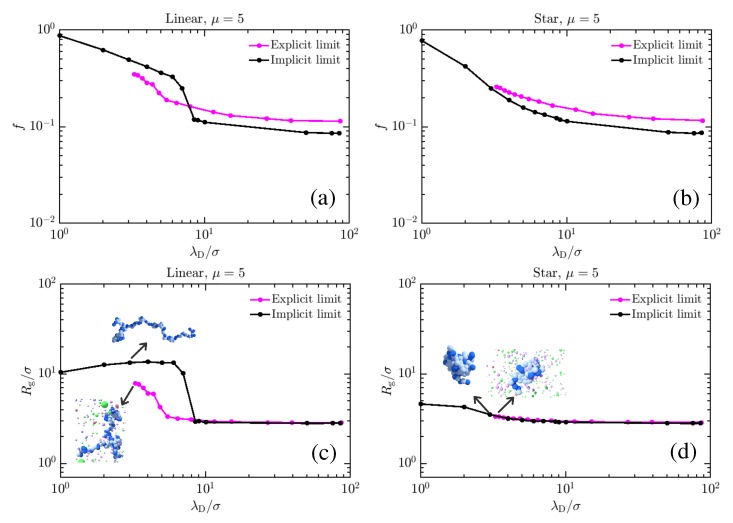
A comparison of the swelling behavior for weak polyelectrolyte chains (**a**,**c**) and stars (**b**,**d**). Panels (**a**,**b**) depict the charge fraction as a function of $\lambda_{D}$ at a fixed μ=5, while (**c**,**d**) plot the corresponding radius of gyration, demonstrating the swelling of the polymers. The plotted $\lambda_{D}$ for explicit-ion systems represents the Debye length calculated from the reservoir ion concentration (see Section 2 for more details). Note that in the case of a linear polyelectrolyte, the charging and swelling effects as $\lambda_{D}$ is decreased are much less pronounced in the explicit salt limit, as screening of the strong charged interactions between adhering monomers is necessary to charge the polymer, which requires ions to enter the collapsed core, energetically favorable, but entropically unfavorable enough to strongly limit swelling. The topological restraints of the star polyelectrolyte (**b**,**d**) cut down the amount of entropy that can be realized in swelling of the polyelectrolyte, thus suppressing charging and swelling significantly in both cases. Further, it should be noted that the charging curves for the explicit-ion systems are remarkably similar in both cases, due to both states remaining largely collapsed until $\lambda_{D}$ is on the order of a monomer diameter. Snapshots in panels (**c**,**d**) correspond to typical configurations, with nearby counterions in the explicit salt case, at the state denoted by the arrow.

First, we analyze the charging of the linear and star weak polyelectrolyte in the implicit and the explicit limit. Figure 3a,b depict the charging behavior (characterized by the fraction of charged monomers *f*), while Figure 3c,d plot the radius of gyration ($R_{g}$) for these polyelectrolytes at μ = 5. Two major effects are notable. First, the star suppresses charging in both cases relative to the linear polyelectrolyte, and with it, suppresses swelling. This is perhaps unsurprising, as the topological constraints enforced by the central bead signify that there is significantly less entropy available to the swollen polyelectrolyte, which shifts the free-energetic balance toward enthalpically favorable adhesion. Second, the explicit ion representation, which initially favors enhanced charging (with ≳10% of monomers charged at all $\lambda_{D}$), actually suppresses charging in both cases for both the linear and star polymers. This may also be understood by appealing to entropic ideas [29,80,81,82,83,84,85,86,87,88]. In this case, the ion entropy is strongly limited when it penetrates the collapsed core of the weak polyelectrolyte in order to take advantage of enthalpically favorable charge interactions, themselves favored due to the value of μ [44]. Adhesive interactions alone are insufficient to collapse the polyion, as evidenced by the swelling of the implicit salt case, suggesting that entropy plays a significant role in determining the state of the polyelectrolyte. This is further corroborated by examining the similarity between the charging and swelling behavior of linear chains and stars in the explicit salt case. The charging curves are remarkably similar until sufficient numbers of ions exist in the system to unlock the polyelectrolyte chain, which happens for $\lambda_{D}$ on the order of the monomer diameter. Linear chains, which can gain more entropy, will swell, while stars do not exhibit significant swelling. This points to a more complex tug-of-war between different influences on the CGT of weak polyelectrolytes than the Debye-Hückel picture can capture. Notably also, while the Debye-Hückel version of the model has a re-collapse at very high monovalent salt concentrations [45,46,47]. we do not see any evidence that this will occur with explicit salt. Indeed, we do not expect it, as it is entropically unfavorable for sufficient numbers of co- and counter-ions to occupy the region around the polyelectrolyte and meaningfully screen charged monomer interactions. The behavior of explicit-salt simulations is thus more similar to what has been observed in experiment [49,66,89].

**Figure 4:**
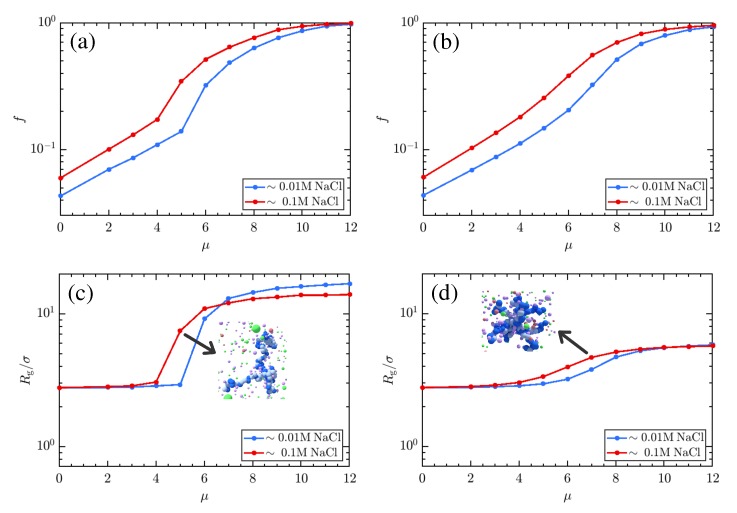
Effects of charging chemical potential μ on the state of weak polyelectrolyte. As in Figure 3, panels (**a**,**c**) plot results for the linear polymer, while panels (**b**,**d**) show the behavior for 10-arm stars. The dependence of charge fraction *f* on μ is given in (**a**,**b**), and each demonstrates clear divergence from the Henderson–Hasselbalch behavior expected of isolated monomers. Adhesive interactions suppress charging of the polyelectrolytes until a salt-concentration-dependent threshold value of μ is reached. Increasing the salt concentration favors charging. Notably, there appears to be a discontinuous jump in the charge fraction *f*, indicative of a first-order phase transition, in the linear polyelectrolyte that is not present for the star. Panels (**c**,**d**) demonstrate the swelling that accompanies the increased charge in each polymer. Swelling is more prominent when the polyelectrolyte is more charged in both cases, though the effect is more pronounced in the linear rather than the star polymer. This can be understood by appealing to relative compactness of each polymer, which disfavors swelling and charging relative to compact conformations which maximize adhesive monomer interactions, and the larger entropy that the linear chain gains in transitioning to a coil relative to that available in the star. Larger salt concentrations permit more ions to occupy the cloud around the polyelectrolyte, increasing both enthalpically favorable binding of counterions, and screening between sites on swollen polymers.

**Figure 5:**
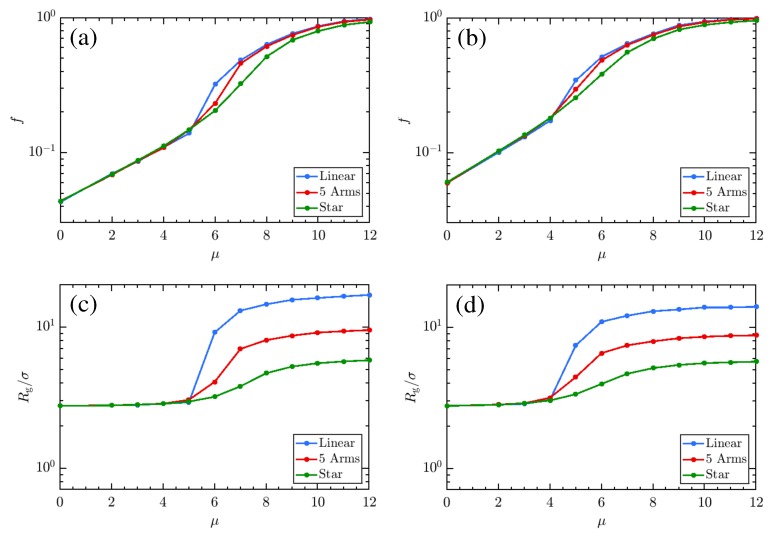
Effect of polymer topology on the CGT behavior of the weak polyelectrolytes. Molecular weight for all topology is identical. (**a**,**c**) represents the low NaCl conditions (∼0.01 M) whereas as (**b**,**d**) represents the high NaCl conditions (∼0.1 M). The figure depicts that the charging and swelling behavior of 5-arm stars is in between that for the linear and 10-arm polymer, indicating *smoothing* of the CGT as we go from linear to 5-arm to 10-arm star polymers, due to increased topological restrictions as the structure of the polymer becomes more branched. Overall, this figure suggests the suppression of charging (deprotonation here) induced continuous CGT with the increase in the number of arms of the polymer.

Figure 3 suggests that important thermodynamics affecting the charge and conformation of weak polyelectrolytes are missed within the implicit-salt representation. It is important, then, to understand how mutual variation of μ and salt concentration manifest in the physical state of weak polyelectrolytes. Knowing this, we proceed to explore how the charging behavior of linear and star polyelectrolyte is affected due to the variation of μ in our explicit-salt representation. In Figure 4 a–d, we show the effects of varying μ at fixed NaCl concentrations, demonstrating a clear preference for increased charging and swelling of the polymer conformations at larger μ for both linear chains (a,c) and stars (b,d). The effects of topology are fairly strong here, and visible in both curves. While a sharp transition from a collapsed to an expanded state (and concomitant jump in charge fraction *f*) occurs for the linear chain, the star is unable to dramatically swell and thus exhibits only a gradual increase in its charge and size. The character of the CGT is thus strongly affected by the confinement, as was previously noted for studies on implicit-salt systems [45]. The effect of increased salt concentrations is to facilitate charging, which in turn facilitates swelling of the chain at small μ, though due to screening effects, swelling is enhanced at low salt concentrations; this is more pronounced for the chain (Figure 4c) than for the star (Figure 4d). Overall, increases in the monovalent salt concentration provide a favorable charging environment (evidenced by the shifting of the CGT transition to lower μ) but limit the extent of swelling. Related results in Figure 5 demonstrate variations in charging behavior for star polymers with different numbers of arms and equal molecular weight. Note that both the charging and swelling behavior of 5-arm stars is between that seen for the linear and 10-arm cases, with a CGT transition that is more gradual (though still reasonably sharp) due to topological restrictions. While the extent of swelling is limited, the additional entropy afforded the 5-arm stars yields curves for *f* with varying μ that are closer to those observed for the linear polyelectrolyte.

**Figure 6:**
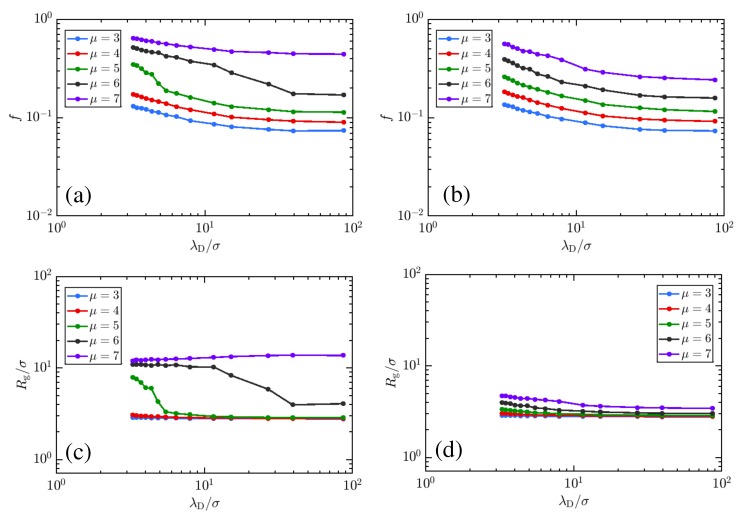
Influence of NaCl concentration on the titration of the linear and star polyelectrolytes for varying μ conditions spanning the CGT. Panels (**a**,**c**) plot the charge fraction *f* and radius of gyration $R_{g}$ for linear weak polyelectrolytes, while panels (**b**,**d**) depict the same quantities for 10-arm star polymers. As intuition would suggest, an increase in μ leads to a higher charge for both linear and star polymer. For linear polymers, the CGT is seen to shift toward lower values of μ as the salt concentration is increased, as evidenced by the gap between higher-charge and more swollen states shifting from between μ=6 and μ=7 at low salt concentrations (high $\lambda_{D}$) to between μ=4 and μ=5 at high salt concentrations (low $\lambda_{D}$). Note that increasing the strength of screening results in higher *f* and reduced $R_{g}/\sigma$ uniformly across μ. For the star polymers in panels (**b**,**d**), a continuous transition in each measured quantity is seen for all μ and $\lambda_{D}$ values.

In Figure 6 we further explore the interplay of μ with salt concentration. There the charge and swelling behavior are plotted for μ values between 3 and 7. A clear shift in the transition for the linear polyelectrolyte is signified by the gap between curves in *f* and $R_{g}/\sigma$ shifting from between μ=6 and 7 to between μ=4 and 5. Suppression of charging in the star polyelectrolyte is observed for high μ. Though the polymers remain more compact throughout, the charge fractions for stars are very close to those for linear chains so long as μ is below the threshold for the CGT, as both types of polymer are compact globules in that regime. Examining the μ=3 curves in Figure 6a,c for the linear polymer shows them virtually indistinguishable from those for the star in Figure 6b,d. This is easily understood, as the charging behavior for compact globules is not significantly different based on connectivity, but for swollen coils, the linear polyelectrolyte can extend much further, enabling significant favorable charging to occur without incurring strong energetic penalties. The more compact star does not have this ability to spread the charges and thus experiences much more modest gains in charge whether it is partially swollen or not. These results are qualitatively similar to previous observations where salt was handled implicitly [45,46]. In comparison to the linear polyelectrolytes, Figure 6b,d suggest a smooth ‘second-order’ CGT transition for star polymers at μ values from 5 to 7 with increase in monovalent salt concentration acting to increase the charging tendency at each μ value, and increase in μ value facilitating charging at all $\lambda_{D}$, as has been suggested in previous experiments [49,89].

The results of Figure 3, Figure 4, Figure 5 and Figure 6 together present an interesting picture regarding the effect of charging chemical potential, monovalent salt concentration, and structure on the titration of the weak polyelectrolyte. These results suggest that, while monovalent salts can act to increase the charging tendency of each monomer, they also act to collapse the polymer chains. Though this is in accord with results from the Debye-Hückel model, the effect is diminished in the explicit salt models due to limits imposed on the local salt concentration by the physical size of ions. One may understand the interplay better by examining a few cases. Examining the plots in Figure 4, we see that at intermediate μ values (For e.g., μ = 5) along with high NaCl concentration ($\lambda_{D}$ ≈ 3σ) the polymers are more swollen, the energetic penalty to charging due to repulsive like-charged neighbor interactions is overcome by the combination of μ and the favorable electrostatic environment provided by solvated counter- and coions. Keeping the chemical potential the same, but lowering the salt concentration ($\lambda_{D}$ ≈ 10σ), the charging penalty and adhesive interactions conspire to yield globular states. For, star polymers, which experience a more substantial energetic penalty to charging through increased confinement of monomers, swelling is suppressed and the effective pK${}_{a}$ for the polymer is shifted. While these also experience a CGT transition, it does not manifest in the same way. The transition in weak stars takes on more of a second-order character, exhibiting a continuously varying behavior in charging and swelling rather than a discontinuous jump. In general, we can take away that the effect of charged monomer repulsion and monomer adhesion are to shift the polymer’s effective pKa to higher pH (from μ=0 to larger values) while the effect of salt, in general, is to shift it back toward the monomeric value. Such shifts come at a cost, as they limit the swelling the polymer undergoes, which is essential for technological applications of pH-driven materials. Topological restrictions shift the effective pK${}_{a}$ even farther away from the Henderson–Hasselbalch expectation, limiting both charging and swelling.

**Figure 7:**
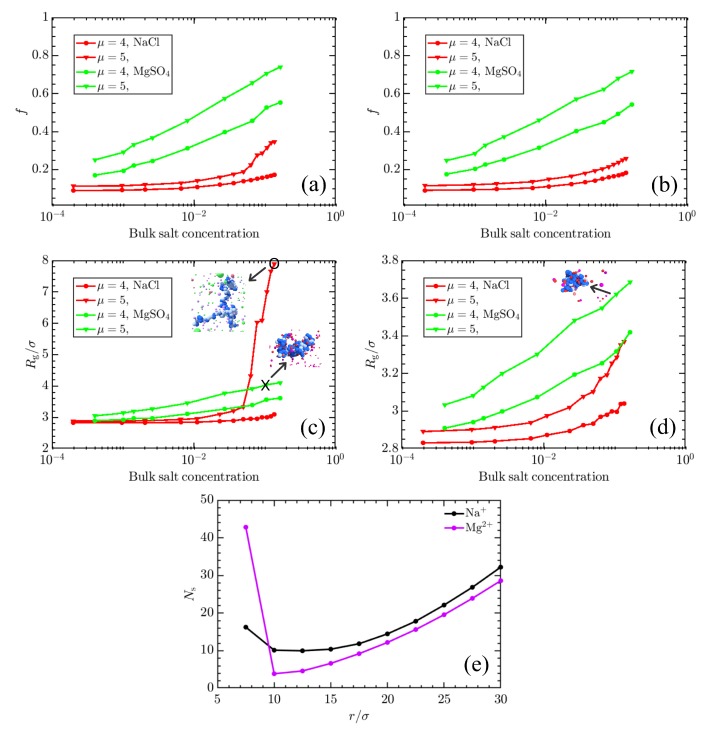
Effect of salt valence on the charging of the weak polyelectrolyte. Panels (**a**,**b**) show the charging behavior of the linear and star polyelectrolyte, respectively, in the presence of monovalent salt (NaCl) and divalent salt (MgSO${}_{4}$) at μ=4 and μ=5. Panels (**c**,**d**) depict the $R_{g}/\sigma$ for linear and star polyelectrolyte respectively corresponding to conditions of (**a**,**b**). An increase in the concentration of MgSO${}_{4}$ leads to higher charge fractions *f* compared to NaCl but retains the collapsed configuration. In order to elucidate the different charging and swelling behavior seen in (**a**–**d**), panel (**e**) depicts the distribution of Mg${}^{2+}$ and Na${}^{+}$ ions, denoted as $N_{s}$, in the concentric spherical shells around the center of mass (CM) of the linear polymer, for the states corresponding to point ’X’ and ’O’ in (**c**).

Finally, we briefly explore the role of ion valence in determining weak polyelectrolyte morphologies. It has been shown in previous studies that ion valence has a significant effect on the CGT of the fully charged strong polyelectrolyte systems [90,91,92] with counterion valences |z|≥2 leading to collapse of strong polyelectrolyte chains and eventual overcharging and reswelling as the concentration of counterions is further increased. While some effects have been examined, there is not yet literature which explicitly addresses the mutual roles of ion valence and topology on the CGT. To probe this limit, we adjusted our model, replacing the monovalent salt reservoir with a divalent MgSO${}_{4}$ reservoir. The divalent salt in our methods does not account for chemical identity and hence can be representative of any divalent salt (MgSO${}_{4}$ differs from NaCl only in ion charge). Figure 7a,b show the effect of divalent salt such as MgSO${}_{4}$ on the charging behavior of linear and star weak polyelectrolyte at μ=4 and μ=5, respectively.

Figure 7 depicts an interesting result regarding the enhancement of the charging of both linear and star polyelectrolyte in the presence of divalent counterions. For example, the charge fraction of the linear polymer in Figure 7a increases from f≈0.35 at μ=5 for NaCl to f≈0.75 for MgSO${}_{4}$. Figure 7c depicts the corresponding $R_{g}$ values and suggests that this increased tendency to charge results from more energetically favorable interactions with divalent ions, which act to collapse, rather than swell, the polymer. This is similar to the collapse of the strong polyelectrolyte chain after neutralization with divalent counterions previously reported [90]. For star polymer, the charging and $R_{g}/\sigma$ follows similar behavior to the linear polymer in the presence of MgSO${}_{4}$ and agrees with the theoretical work by Nap and co-workers, where Ca${}^{2+}$ concentration-dependent collapsing of end-tethered poly-acrylic acid (PAA) was suggested [93].

Some significant conclusions can be made from the results presented in Figure 7. First, the linear weak polyelectrolyte undergoes the CGT with increasing concentration of monovalent salt such as NaCl at intermediate μ values, but this does not happen for multivalent salt even though the observed charge *f* is higher with MgSO${}_{4}$ present. Secondly, the topology of the weak polyelectrolyte (see Figure 7b,d) does not play a role in the charging behavior with MgSO${}_{4}$, likely because swelling is not pronounced for these systems. Neutralization via divalent counterions has been known to result in collapsed or lower $R_{g}$ structures [94,95,96,97], which explains why globular structures are indicated even at conditions of high polymer charge in the presence of MgSO${}_{4}$ in Figure 7. The distribution of counterions shown in Figure 7e corroborates this, showing a high concentration of MgSO${}_{4}$ counterions near the polymer (r<7.5σ) suggesting divalent counterion condensation on the polymer chain, contributing to neutralization and collapse. This phenomenon of increased ionization of the weak polyelectrolyte along with condensation of the divalent counterion on the polymer chain was also observed in work done by Qu et al. [66], where ionization along with SO${}_{4}^{2-}$ condensation on P2VP chains was reported. In the case of NaCl, a comparatively diffuse distribution is seen, with ions acting primarily to screen the charge. This behavior of weak polyelectrolytes depicted here in the presence of divalent salt could be particularly useful for adhesion of polymers in hydrogels and can offer more tuning opportunities for targeted drug delivery [96,98,99,100,101,102].

## 4. Conclusions

We have presented a comprehensive picture of titration of weak polyelectrolytes taking into account the explicit pH and salt effects. The results indicated that the implicit Debye-Hückel model is not able to properly take into consideration entropic effects due to ion–monomer interaction, especially at high monovalent salt concentrations, where the most notable differences in the implicit and explicit limit were noted. Furthermore, we were also able to characterize the effect of polymer structure on the ionization extent as observed in experiments [49] and depict the weak polyelectrolyte collapse, in corroboration with experiments [66], similar to that observed in strong polyelectrolytes [91] in the presence of divalent salt even though the ionization degree of the weak polyelectrolyte was much greater in comparison to the monovalent salt, where, instead swelling of the polyelectrolyte was observed. Again, divalent salt effects could not have been accounted in the implicit Debye-Hückel model further emphasizing the importance of including explicit salt to elucidate weak polyelectrolyte behavior. Importantly, we can observe and explain several effects that are corroborated by comparison to experiments.

In cases where the architecture of the weak polyelectrolyte is modified, one can infer some effects on the polymer’s titration behavior based on the results presented here. For example, one might be interested in the titration behavior expected if the arm length of the weak star polyelectrolyte, investigated in this study, is increased by the addition of monomers. As sites become more removed from the central core of the star, their local environment more closely resembles sites in a long linear chain, and the charging behavior of the polymer as a whole would be increasingly dominated by these sites. Hence, we would expect the CGT to revert to more of a first-order-like transition there, provided the arms are long enough. Conversely, if the approximate arm length is kept the same, but the number of branches is increased, yielding a higher number of centrally tethered arms or a dendritic polymer with a known average number of monomers between branch points, the suppression effects are enhanced, and should increase in importance as the density of branch points is increased. One would expect similar charge suppression in polyelectrolyte brushes (Where a high density of polymers is grafted to a surface) and in polyelectrolytes which are confined and cannot separate from each other. This prediction could be tested experimentally by examining the swelling behavior of weak polyelectrolyte brushes with known density or dendritic polymers with known cross-linking density. One important concept to note is that if other high-density ions are present, charging is enhanced, and the polyelectrolytes concomitantly collapse. This effect is similar to what was observed in the divalent ion simulations of this work, and what has been examined for weak polyelectrolyte interactions with oppositely charged polyelectrolytes or nanoparticles [33,57,62,103].

While it should be noted that the weak polyelectrolytes in this study are coarse-grained, and ignore some potentially significant effects such intra- and intermolecular hydrogen bonding, dipolar interactions, charge delocalization and realistic polymer backbones, the work here nevertheless provides a stepping stone to investigating weak polyelectrolyte behavior in different applications such as in polyelectrolyte complexation [33], coacervates [28], and nanofiltration membranes [15]. The properties of weak polyelectrolytes in dense brush systems and polyelectrolyte complexes where water is more sparingly present constitute a frontier of polyelectrolyte physical chemistry which the techniques and analyses presented in this study can begin to explore and explain.
